# Esophageal pressures in supine and prone position: evaluation using high-resolution manometry

**DOI:** 10.1007/s10877-026-01456-6

**Published:** 2026-06-11

**Authors:** Viktor Erbring, Magni Gudmundsson, Hannes Widing, Sophie Lindgren, Christian Rylander, Per Persson

**Affiliations:** 1 Department of Anaesthesiology and Intensive Care Medicine, Institute of Clinical Sciences, Sahlgrenska Academy, University of Gothenburg, Gothenburg, Sweden; 2 Department of Anaesthesia and Intensive Care, Region Västra Götaland, Sahlgrenska University Hospital, Gothenburg, Sweden; 3 Anesthesiology and Intensive Care, Department of Surgical Sciences, Uppsala University, Uppsala, Sweden; 4 Department of Cardiothoracic Anaesthesia and Intensive Care, Sahlgrenska University Hospital, Gothenburg, 413 45 Sweden

**Keywords:** Prone position, Esophageal pressure, Transpulmonary pressure, Lung injury

## Abstract

Esophageal pressure (PES), used as a substitute for pleural pressure in calculations of respiratory mechanics, are complex and influenced by several factors. High-resolution manometry (HRM) provides pressure readings over the esophageal length, which makes it possible to identify factors other than respiratory mechanics that influence esophageal pressure. The aim of this study was to describe and explore esophageal pressure in prone and supine position at different ventilatory settings. PES was measured using HRM in 20 mechanically ventilated patients before start of spinal surgery. PEEP of 5 and 12 cmH_2_O were used in supine and prone position while data were recorded continuously. Mean end-expiratory (PES_EE_) and tidal variation of esophageal pressures (ΔPES) from sensors in different esophageal regions were compared including a selection of sensors with theoretically optimal pressure readings (PES_OPT_). PES_EE_ was significantly lower (MD 3.2–5.7), ΔPES was significantly higher (MD 0.8–1.6 cmH_2_O) and cardiac oscillations were significantly smaller in prone compared to supine position. Differences were dependent on PEEP level and regions of esophagus included. Mean within patient variability of PES_EE_ and ΔPES was 38–540% depending on PEEP level and body position. End-expiratory PES_OPT_ was lower than mean PES_EE_ from larger esophageal regions and less variable. A large variation in PES is seen in both supine and prone position but depends on which sensors are included in calculations of PES. There are significant differences in absolute pressures and in influence from mediastinal organs between supine and prone position.

**Supplementary Information:**

The online version contains supplementary material available at https://doi.org/10.1007/s10877-026-01456-6.

## Introduction

Measurements of esophageal pressure, introduced over 100 years ago [1] has been used for evaluation of respiratory mechanics during mechanical ventilation for decades [2]. As a substitute for pleural pressure, esophageal pressure enables calculation of the distending pressure of the lung, the transpulmonary pressure [3]. Despite extensive research over decades and the theoretical utility, esophageal pressure measurements was rarely used 10 years ago [4], and has still not been adopted as a standard in the clinical setting in the ICU, even in patients with severe respiratory failure.

Correct interpretation of the pressure obtained from an esophageal ballon catheter, which is the technique primarily used, demands an understanding of not only the relation between esophageal and pleural pressure but also other factors influencing the measured pressure. Extensive evaluation shows that both elements associated with the measurement technique (balloon size [5], balloon position [6] and filling volume of the ballon [7]) as well as external factors other than chest wall mechanics (mediastinal weight, cardiac artefacts, esophageal elastance [8]) are important. The complexity involved in the estimation of pleural pressure from an esophageal balloon likely contributes to the low usage of the technique in clinical practice.

In a previous publication our research group introduced high-resolution manometry (HRM), routinely used in gastroenterology [9], for esophageal pressure measurements during mechanical ventilation [10]. HRM provides separately measured pressures along the esophagus and avoids some of the disadvantages associated with the balloon catheter. The technique enables evaluation of the influence from factors other than respiratory mechanics. In our previous HRM-study the large variability in esophageal pressure within a patient [5] and the effect of posture [11] previously described were confirmed but also shown to be interdependent and affected by ventilator settings [10].

Prone position has been shown to increase survival in mechanically ventilated patients with severe ARDS [12, 13]. The more favorable outcome is thought to be partly explained by a more evenly distributed lung stress caused by changes in lung- and chest wall mechanics [14]. Still, studies evaluating respiratory mechanics with esophageal pressure in prone position are scarce, only performed with a ballon catheter and showing heterogenous results [15–17].

The overall aim of the present study was to explore esophageal pressures in a clinical setting in both prone and supine position with different ventilator settings. An important part was to identify factors not obviously related to respiratory mechanics, that influence esophageal pressure and thereby its relation to pleural pressure, and to determine how these factors are affected by prone position and changes in ventilator settings. A further aim was to compare obtained esophageal pressure to suggested “optimal” esophageal pressure calculated from selected pressure readings.

## Methods

### Patients

In this prospective exploratory physiological study, patients scheduled for elective spine surgery between October 2020 and April 2022 were screened for inclusion. Exclusion criteria were lung disease contraindicating adjustments of positive end-expiratory pressure (PEEP) according to protocol, esophageal disease, BMI > 40 and ASA classification *≥* 3. The study was conducted at Sahlgrenska University Hospital, approved by the Swedish Ethical Review Authority (Ref 2019–06583) and registered in the regional research database, https://www.researchweb.org/is/vgr/, id 277,552. Informed consent was obtained from all patients before inclusion. As this was an exploratory physiologic study, without possibility to extrapolate our possible findings, no formal sample size calculation was performed.

Also, a separate cohort from a previous study with HRM (still unpublished) was included for analysis of the representability of the mean esophageal pressure calculated from groups of pressure sensors in different regions. Airway driving pressure (ΔPAW) and tidal variation of esophageal pressure (ΔPES) was recorded during an occlusion test [18]. This cohort included ten patients in the weaning phase from mechanical ventilation in the intensive Care Unit (ICU), for more details see Table S10 in supplementary material.

### Measurements

Esophageal pressure (PES) was measured with a 2,75 mm diameter HRM catheter (Sierra Scientific Instruments, Inc, Los Angeles, CA, USA) with 36 pressure channels, each fed by 12 circumferential pressure sensors and interspaced by 1 cm (in total 432 pressure sensors). After calibration of all pressure sensors from 0 to 250mmHg in a pressure chamber, according to recommendations from manufacturer, the HRM catheter was advanced to approximately 48 cm from the nostrils to allow separate measurements of pressure along the full length of the esophagus. At the end of the protocol the catheter was withdrawn, and previously calibrated pressures were checked to detect a potential pressure drift, that was later adjusted for by the software, see below. Airway pressure and tidal volume were continuously measured by the Flow-i anesthesia machine (Maquet Critical Care, Solna, Sweden) and recorded by a Maquet dedicated software. If signs of malfunction or erroneous calibration of a sensor, the pressure from that sensor was replaced by the mean of the adjacent functional sensor 1 cm cranial and 1 cm caudal during analysis of the data.

### Protocol

**Fig. 1:**
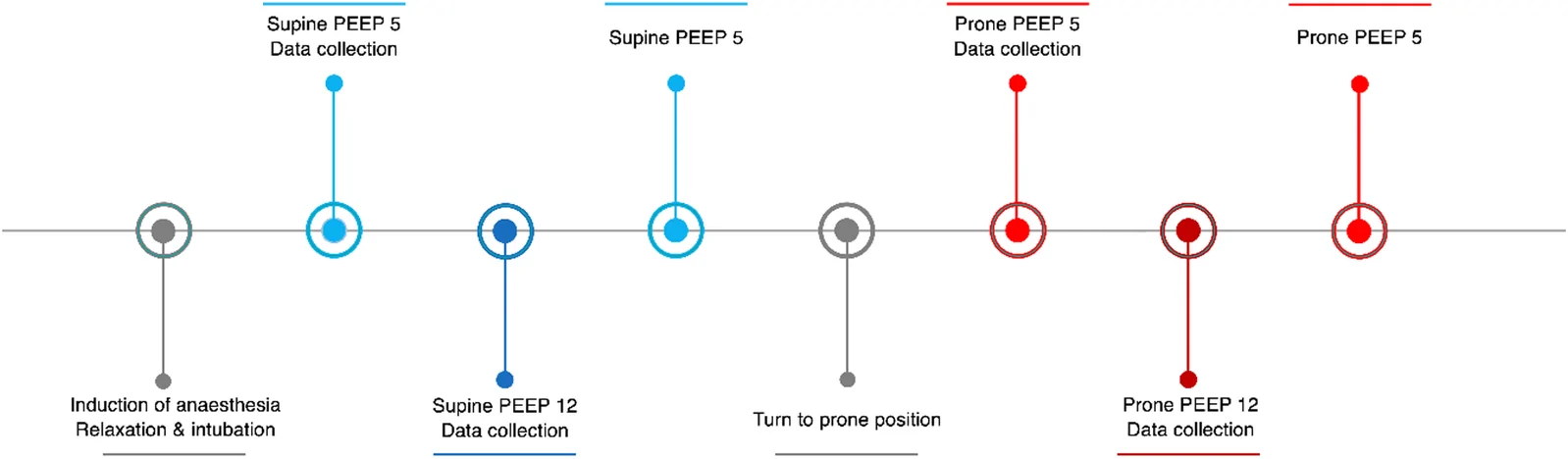
Timeline of the study protocol

Patients were anaesthetized, paralyzed and intubated using propofol, remifentanil, sevoflurane and rocuronium bromide according to standard anesthetic protocol. Train of Four testing (TOF) was performed to ensure adequate muscle relaxation before the start of data collection. At baseline, the patients were positioned supine and ventilated in volume control, PEEP 5 cmH_2_O and tidal volumes of 6 ml/kg ideal body weight. After insertion of the HRM catheter, continuous recording of PES, airway pressure and tidal volumes was initiated. After recording during 20 breaths at baseline, PEEP was set at 12 cm H_2_O [10], and a new recording was made. The PEEP was once again set at 5 cm H_2_O, the patient was then turned prone and after a stabilization period of a minimum of 10 min the same recording at both PEEP levels was repeated (Fig. 1). Pressures from all 36 pressure channels were recorded continuously and available for analysis.

### Analysis and calculations

During the analysis, data from every second pressure channel from 22 to 44 cm from the nostrils were included in analysis, in total 12 pressure channels. Pressures from > 44 cm from nostrils are below the recommended position for a traditional balloon catheter and are at increased risk of influence by the lower esophageal sphincter. In order to avoid influence from peristaltic contractions, mean PES from 5 consecutive stable breaths at each body position and PEEP level was used in calculations, analyzed data was collected after a minimum of 15 breaths after PEEP adjustment. Tidal variation of esophageal pressure (ΔPES) was computed at each level as the difference between end-inspiratory and end-expiratory PES (PES_EI_-PES_EE_). Mean pressure from all 12 included pressure channels 22–44 cm from nostril (PES_22−44_), from the 7 channels 30–42 cm from nostrils (PES_30−42_), which is the region recommended for balloon positioning, and from selected channels (PES_OPT_), see below, was calculated and are presented separately.

Cardiac oscillations were used as a marker of external influence of mediastinal organs on esophageal pressure. The sensor displaying the largest cardiac oscillations was identified in supine position. The magnitude of oscillation at that level in supine and prone at both PEEP levels was compared.

Based on the finding of large variations in both PES_EE_ and ΔPES in our previous study [10], and studies indicating that PES generally overestimates pleural pressure [2, 11, 19] a separate analysis of PES from selected esophageal pressure channels, potentially best representing the pleural pressure, was performed. This “optimal esophageal pressure” (PES_OPT_) was defined as the mean pressure from the three pressure channels in the lower esophageal region (30–42 cm from nostrils) with the lowest end-expiratory esophageal pressure (PES_EE_) and a positive ΔPES during tidal inflation. Analysis of the representativity of PES_22−44_, PES_30−42_ and PES_OPT_, was performed by comparing the ratio of mean ΔPES from included pressure channels and ΔPAW in a separate cohort.

### Statistics

All data are presented as mean (SD) and median (min; max) for continuous variables and n (%) for categorical values. The mean esophageal pressure in a patient was calculated as the mean from sensors in a specified area and presented with the within patient standard deviation. Esophageal pressure differences between patients were normally distributed as assessed by histogram and Shapiro-Wilks test. Differences are presented as mean with 95% confidence intervals, intervals separated from zero are considered statistically significant. Intraindividual variation is evaluated using within patient SD, coefficient of variation (calculated as SD/mean PES) and comparisons of highest and lowest PES (PES_MAX_-PES_MIN_ within a patient).

## Results

### Patient characteristics and ventilatory data

20 patients were included, 11 (55%) female, age 55.7 (17) years, BMI 27.5 (4.9) kg/m^2^. During data recording tidal volumes were 6.0 (0.1) ml/kg IBW. PEEP was 5.1 (0.3) and 12.2 (0.2) cmH_2_O in supine position, and 5.2 (0.2) and 12.1 (0.2) cmH_2_O in prone position, Table 1 and supplementary material table S1.

**Table 1:** Patient characteristics and baseline ventilator data

Age (years)	Sex	ASA	Height (m)	Weight (kg)	BMI (kg/m2)	IBW (kg)	VT (ml)	VT (ml/kg IBW)
73	F	2	1.6	60	23.4	52	320	6.15
65	F	2	1.69	91	31.9	61	370	6.07
62	M	2	1.73	80	26.7	69	420	6.09
62	M	2	1.75	100	32.7	70	420	6.00
57	F	1	1.71	64	21.9	62	370	5.97
76	M	2	1.87	88	25.2	81	490	6.00
21	F	1	1.62	60	22.9	54	330	5.81
57	M	2	1.75	80	26.1	70	420	6.00
53	F	2	1.63	92	34.6	55	330	6,00
61	M	2	1.79	85	26.5	74	450	6.08
68	M	2	1.9	84	23.3	84	500	5.93
71	M	3	1.76	80	25.8	71	430	6.06
66	M	3	1.78	78	24.6	73	440	6.00
76	M	3	1.84	100	29.5	81	490	6.02
59	F	2	1.72	80	27	63	380	5.95
56	F	2	1.61	100	38.6	53	320	6.02
39	F	2	1.7	102	35.3	61	370	6.03
17	F	1	1.66	57	20.7	58	350	6.00
33	F	1	1.76	75	24.2	67	400	5.93
42	F	1	1.8	94	29	70	420	5.94

### Esophageal pressure with HRM

**Fig. 2:**
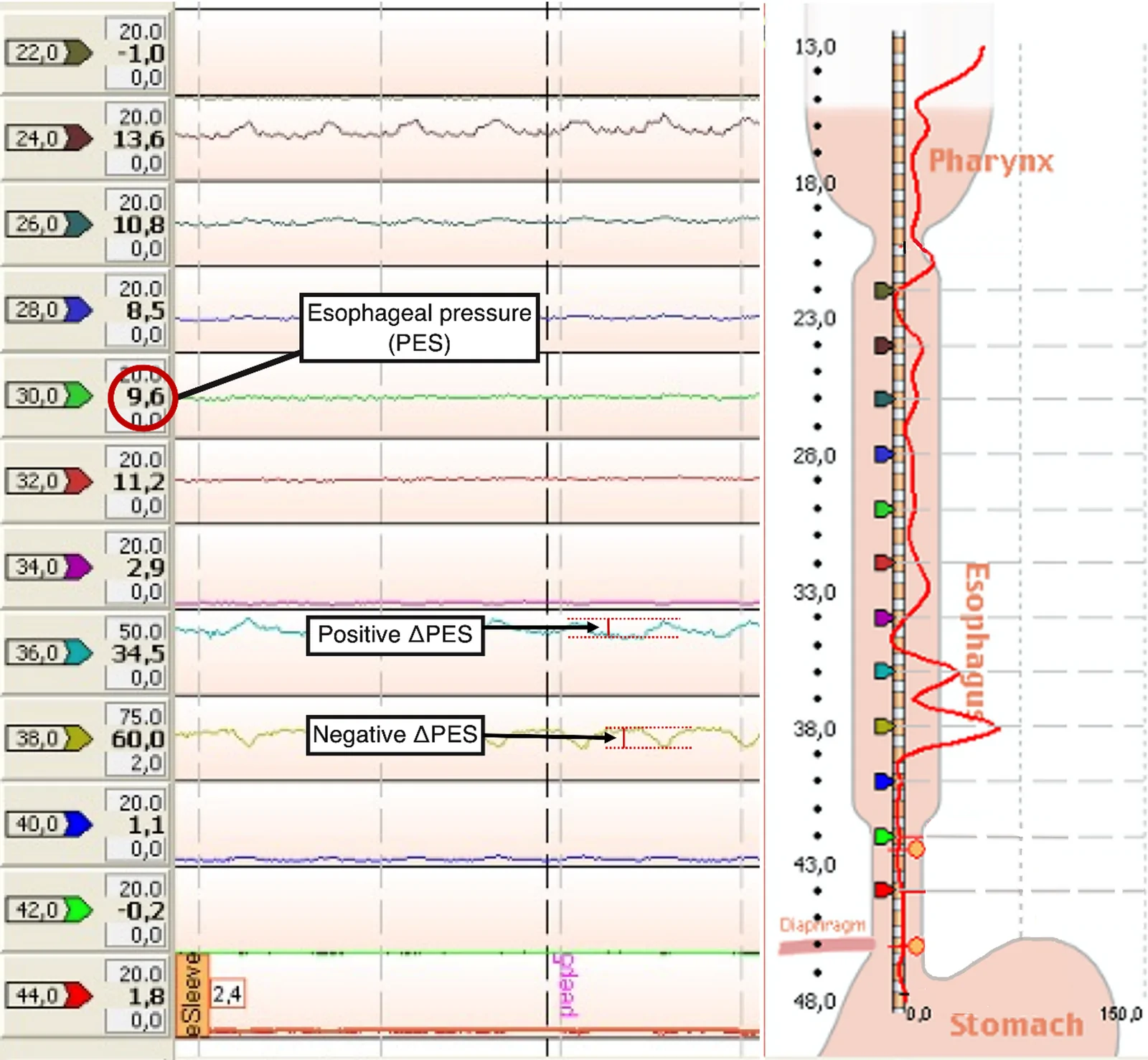
Example of esophageal pressures from patient 10 in prone position, PEEP set to 5. Pressure presented in mmHg. Level of selected pressure sensors 22–44 cm from nostril are seen on the left

HRM measurements were successfully performed in all patients without complications. In two patients, pressures from a total of three sensors needed to be replaced due to sensor malfunction or erroneous calibration. For a representative measurement of esophageal pressure with HRM see Fig. 2. When pressure readings were analyzed according to criteria for optimal PES in our cohort, less than three sensors fulfilled the criteria, and therefore pressures from fewer than three sensors were used for analysis in two patients. In the separate cohort (previously described), occlusion test (Baydur maneuver) was performed. ΔPES/ΔPAW ratio was 0,89, 1.08 and 1.12 for PES_22−44_, PES_30−42_ and PES_OPT_ respectively. See table S2 in supplementary file.

### End-expiratory esophageal pressure (PES_EE_)

**Fig. 3:**
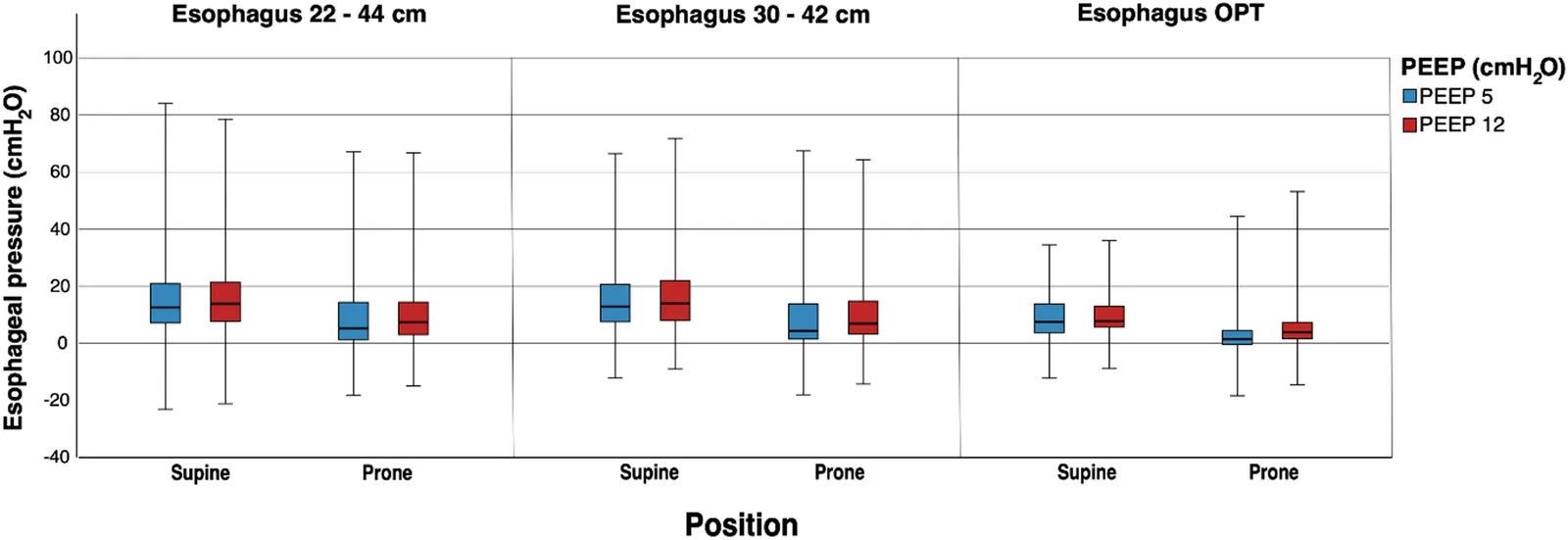
Median end-expiratory esophageal pressure in supine and prone position. Calculated from selected pressure sensors: PES_22−44_, PES_30−42_ and PES_OPT_. Horizontal line represents median value, boxes represent interquartile range and whiskers min max values

There was a significant difference in PES_EE_ in supine compared to prone position with PES_EE_ being 3.2–5.7 cmH_2_O lower in prone depending on part of esophagus and level of PEEP, Table 2; Fig. 3. End-expiratory PES_OPT_ was 5.3–6.6 cmH_2_O lower than PES_30−42_, depending on body position and PEEP-level, see table S3 in supplementary file.

Mean PES_EE_ significantly increased when PEEP was increased in both supine and prone position, except for the mean PES_22−44_ cm in supine position. The change in PES_EE_ in response to an increase in PEEP was significantly higher in prone position, mean difference (95% CI) for PES_30−42_ 0,9 (0,1; 1,7), PES_22−44 cm_ 0,8 (0,0; 1,5) and PES_OPT_ 2,1 (0,7; 3,4) cmH_2_O respectively.

For presentation of median end-expiratory pressure in all patients divided dependent of region of esophagus included, see Fig. 3.

**Table 2:** Mean PES_EE_ and ΔPES in supine and prone at different PEEP-levels. All pressures presented in cmH_2_O

	End-expiratory esophageal pressure (PES_EE_) in cmH_2_O, *n* = 20								
	Supine 22–44 cm Mean (SD) Median (min; max)	Prone 22–44 cm Mean (SD) Median (min; max)	Difference Mean (95% CI)	Supine 30–42 cm Mean (SD) Median (min; max)	Prone 30–42 cm Mean (SD) Median (min; max)	Difference Mean (95% CI)	Supine PES_OPT_ Mean (SD) Median (min; max)	Prone PES_OPT_ Mean (SD) Median (min; max)	Difference Mean (95% CI)
PEEP 5	15.9 (7.9) 14.1 (7.8;38.4)	10.2 (10.3) 6.7 (1;40.8)	5.7 (2.3; 9.1)*	15.6 (8.7) 14.5 (5.4;38.5)	10.2 (11.5) 5.1 (-0.5-40.7)	5.4 (1.2; 9.6)*	9.5 (7.6) 7.2 (1.4; 28.1)	4.2 (8.8) 2 (-5.6; 37)	5.3 (2.5; 8.1)*
PEEP 12	16.3 (7.9) 14.9 (6.6;40.1)	11.3 (9.9) 9.4 (0.6;43.4)	4.9 (1.6; 8.3)*	16.5 (8.6) 15.3 (3.3;40.6)	11.9 (10.9) 8.1 (-1;43.3)	4.5 (0.5; 8.6)*	9.9 (7.8) 7.4 (0.5; 29.9)	6.7 (10) 4.3 (-2.4; 44.4)	3.2 (0.1; 6.4)*
Difference Mean (95% CI)	0.4 (-0.2; 1.0)	1.2 (0.2; 2.2)*		0.8 (0.0; 1.6)*	1.7 (0.5; 2.9)*		0.4 (-1; 1.8)	2.5 (1.3; 3.6)*	
	Tidal variation of esophageal pressure (ΔPES) in cmH_2_O, *n* = 20								
PEEP 5	1.3 (0.7) 1.4 (0.2;2.5)	2.2 (2.0) 2.4 (-2.4;5.5)	0.9 (0.0; 1.7)*	1.4 (1.2) 1.2 (-0.3;4.1)	3.0 (2.5) 3.4 (-1.8;6.9)	1.6 (0.6; 2.5)*	2.4 (1.4) 2.4 (0.3; 5.9)	3.6 (2.1) 3.5 (0.1; 7.1)	1.2 (0.3; 2)*
PEEP 12	1.6 (0.8) 1.7 (0.4;2.9)	2.4 (2.1) 2.5 (-1.7;6.7)	0.8 (0.0; 1.6)*	1.8 (1.3) 1.8 (-0.5;4.1)	3.2 (2.6) 3.5 (-1.6;7.3)	1.4 (0.5; 2.3)*	2.1 (1.3) 2.1 (0.1; 4.9)	3.4 (2.1) 3.7 (0.1; 6.9)	1.3 (0.5; 2.2)*
Difference Mean (95% CI)	0.3 (0.0; 0.6)*	0.3 (-0.0; 0.6)		0.4 (-0.1; 0.9)	0.2 (-0.1; 0.5)		-0.3 (-0.8; 0.2)	-0.1 (-0.4; 0.2)	

*Confidence interval separated from 0

### Tidal variation of esophageal pressure (ΔPES)

In prone position ΔPES was significantly higher when compared to supine at the same PEEP-level. This effect was most apparent when in the lower esophageal regions (30–42 cm) where ΔPES in prone was 1.6 cmH_2_O (0.6; 2.5) higher at PEEP 5 and 1.4 cmH_2_O (0.5; 2.3) higher at PEEP 12.

**Fig. 4:**
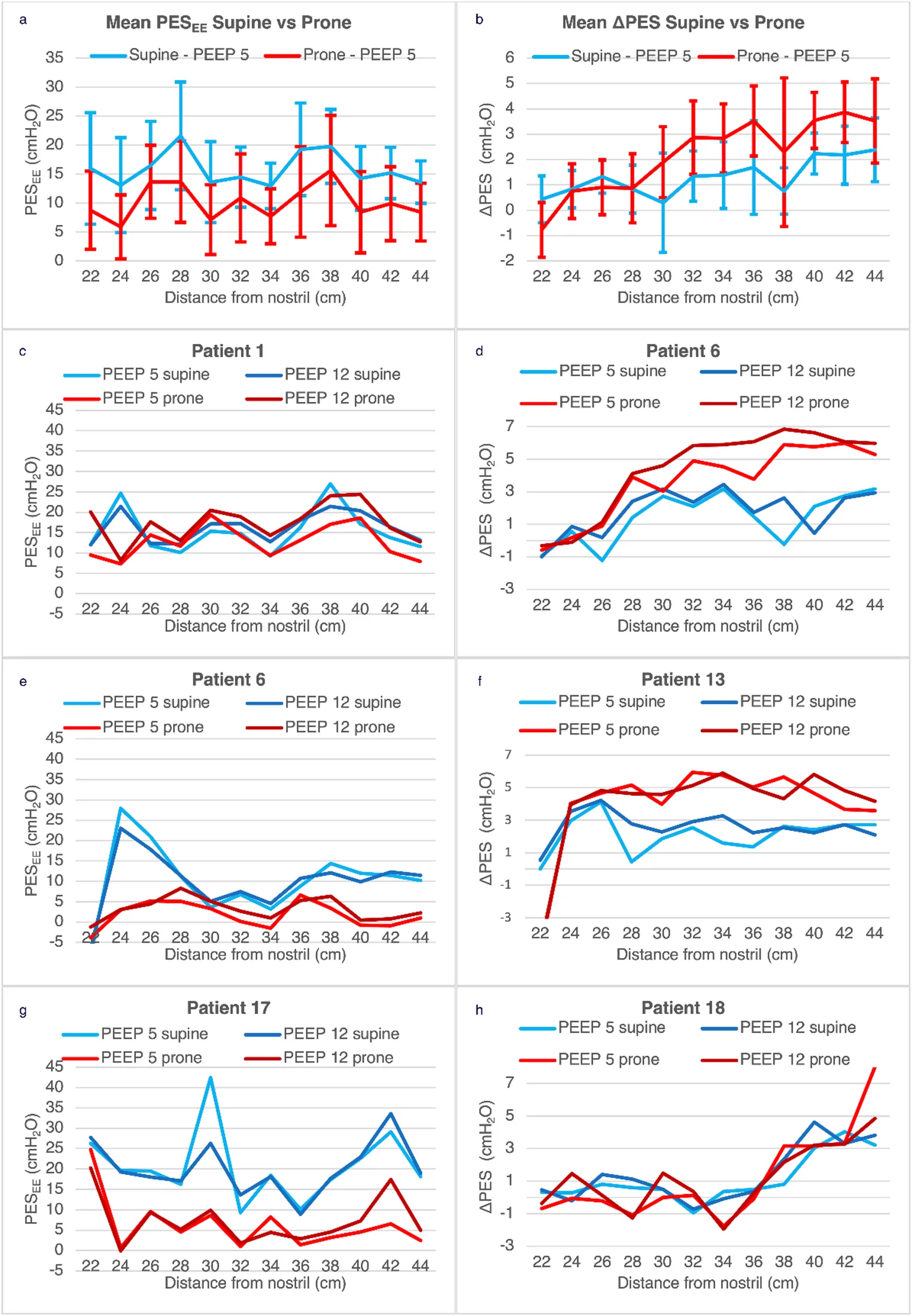
Top left (**a**): Mean PES_EE_ at different levels of the esophagus, brackets representing CI 95%. Top right (**b**): Mean ΔPES at different levels of the esophagus, brackets representing CI 95%. Left column 3 lower figures (**c, e, g**): Individual examples showing PES_EE_. Right column 3 lower figures (**d, f, h**): Individual examples showing ΔPES

An increase of PEEP resulted in a small increase in ΔPES in both supine and prone, except for PES_OPT_. However, the increase was only significant in supine position when calculating from PES_22−44_ (Table 1). ΔPES was negative in ≥1 pressure channel in 18 of the 20 patients in either supine or prone position and in 15 of the 20 patients in both supine and prone position, see Figs. 2 and 4 and fig S1 in supplementary file.

### Variability of esophageal pressures

Esophageal pressure varied within all patients but to different degrees, see Fig. 3 for representative patients and Fig. S1 in supplementary file for all patients. Mean coefficient of variation within esophagus 30–42 cm from nostril was 58%–540%, median 44%–120%. (Table 3). For data on PES_22−44 cm_ and PES_OPT_ see table S4 and S5 in supplementary file.

**Table 3:** Within patient variation of PES_30−42_

	Coefficient of variation 30–42 cm, *n* = 20			
	PESEE		ΔPES	
	Supine Mean (SD) Median (min-max)	Prone Mean (SD) Median (min-max)	Supine Mean (SD) Median (min-max)	Prone Mean (SD) Median (min-max)
PEEP 5	0.63 (0.3) 0.52 (0.3–1.2)	1.1 (1.9) 0.89 (0.3–7.7)	5.4 (10.3) 1.2 (0.2–37.4)	1.13 (1.3) 0.54 (0.1–5.1)
PEEP 12	0.58 (0.3) 0.44 (0.2–1.1)	0.84 (0.6) 0.79 (0.2–2.6)	1.3 (1.2) 0.9 (0.2–4.6)	1.46 (2.9) 0.4 (0.1–12.3)

The mean intraindividual difference between the highest and lowest end-expiratory PES_30−42_ cm from nostril in supine and prone position was 21.9 and 25.0 cmH_2_O respectively and the within patient SD was 8.0 and 8.9 cmH_2_O respectively. The difference between highest and lowest end-expiratory PES_OPT_ was 5.8 in prone and 7.8 cmH_2_O supine position and within patient SD 3.2 and 4.1 cmH_2_O respectively, see table S6 and figure S2 in supplementary file. Variation between patients occurred mostly in the same level as variation within patients, table S7 in supplementary file.

### Cardiac oscillations

**Fig. 5:**
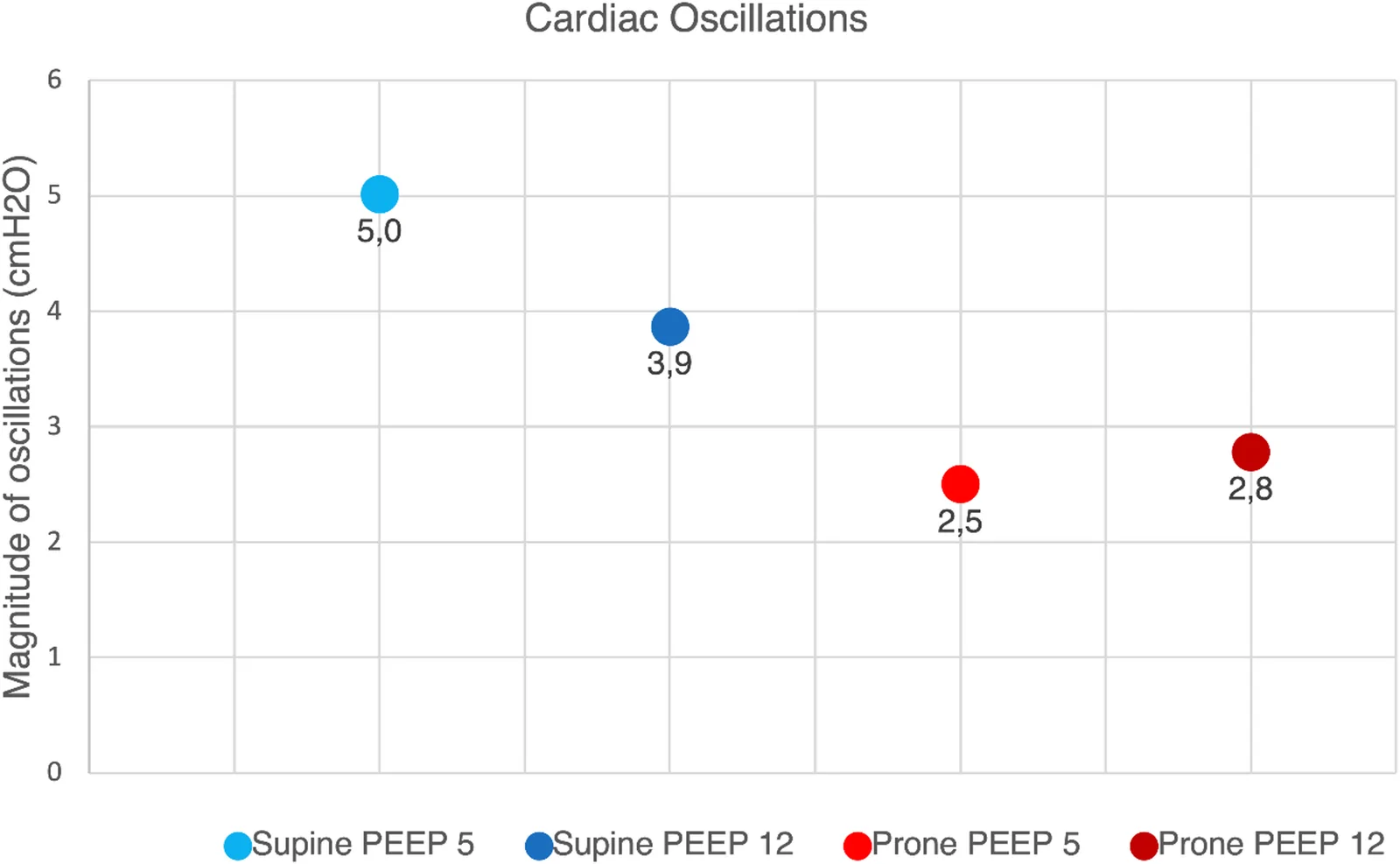
Magnitude of oscillation in cmH_2_O presented as mean

Cardiac oscillations are most prominent at low PEEP in supine position and are significantly affected by PEEP in supine position, decrease of 1.1 cmH_2_O (95% CI 0.1; 2.2), and change to prone, decrease of 2.5 cmH_2_O (1.4; 3.7) Contrary, in prone position cardiac oscillations are not affected by PEEP, difference between PEEP 12 and 5 was 0.3 cmH_2_O (95% CI -0.5; 1.0), see Fig. 5.  

## Discussion

The main results of this study were that esophageal pressures are affected by body position and are highly variable within a patient. This study thereby confirms the main findings in our previous study [10] but also adds further knowledge that compared to supine position, esophageal pressure in prone position remains highly variable, but seems less affected by the weight from mediastinal organs and responds differently to a change of PEEP. This is to our knowledge first study evaluating esophageal pressure in supine and prone position using HRM. End-expiratory esophageal pressure from selected pressure channels in the lower part of esophagus (PES_OPT_) are theoretically less affected by confounding factors and thereby possibly more representable of pleural pressure. These pressures differ significantly from the mean pressure within the region of esophagus where the esophageal ballon is most often placed in clinical practice, PES_30−42_ (Fig. 5).

### Strength and limitations

Since previous evaluations of esophageal pressure rarely include prone position and almost unexceptionally have been performed with a balloon catheter, often 10 cm in length, presenting one pressure affected by the filling volume [7] and position of the balloon [6] this study adds important knowledge. High-resolution manometry provides a unique possibility to understand esophageal pressure by showing a detailed picture of esophageal pressure while avoiding some of the drawbacks with balloon catheters. Still, since the HRM catheter uses solid-state pressure sensors it is subject to pressure and thermal drift, [20–22] which are dependent on the length of the measurement and of the thermal difference between the air and the esophagus. In order minimize these effects we used a catheter that has been previously used in multiple examinations, we had a short protocol and applied the recommended thermal compensation before analysis [22]. The focus on the current study was to assess changes in pressure during interventions and difference between different part of the esophagus and not the absolute pressures per se. Using the HRM method bedside in the ICU is possibly feasible but demanding, however in a study design we can use its advantages to exclude regions which probably aren’t reflecting the actual pleural pressure. The major limitation of this exploratory descriptive study is the low number of patients and that measurements were performed in lung healthy patients, which should be kept in mind when applying our findings to patients with respiratory failure in the ICU. Another difficulty associated with this measurement is to determine to what degree the PES is representative of pleural pressure, or a result of other factors. The unpredictable variability with no obvious recognizable pattern between patients makes it hard to determine exactly what factors are responsible for causing local high pressures and the high variability within a patient.

### End-expiratory esophageal pressure

Suggested to be representative of pleural pressure [23], the end-expiratory esophageal pressure has been used as a basis for ventilator settings, mainly PEEP [24]. To understand the rationale behind this view of the absolute end-expiratory esophageal pressure is challenging when provided with the more detailed picture in this and our previous study [10]. The large variability in end-expiratory pressures within a patient, often within 2 cm of distance, in both supine and prone position unlikely represents an equal variation of pleural pressure but rather an influence from external forces other than the chest wall. The vertical gradient of pleural pressure caused by the lung weight, 0.3-1.0 cmH_2_O/cm depending on measurement technique, is well known [25] but very different from the variation described above and in other studies [5, 9].

Local high pressures within the esophagus (> 30 cmH_2_O) are seen in a majority of patients in this study and was also a common finding in our previous study using HRM [10]. All sensors on HRM are calibrated before insertion and checked afterward, see method, and presented pressures are unlikely explained by measurement errors. High pressures at the end-zones of the esophagus, can possibly be explained by the upper and lower esophageal sphincter [26] but high pressures are also seen in other areas of esophagus. Disparities between esophageal pressures from “catheter-mounted” transducers and balloon catheters are well-known and the occasional higher pressures from the catheter-mounted transducers have been attributed to several factors including mucus on the sensors [27, 28]. The local high pressures seen in this study, are stable over time and the pattern is stable over changes in ventilator settings (different PEEP). In our previous study comparing esophageal pressures in supine and sitting position using HRM, local high pressures are lacking in sitting position but appears when patient is supine [10]. A large variation in esophageal pressure can also be seen when a short ballon is used in different parts of the esophagus [5]. Our conclusion is that the large variability in pressures, including high pressures, exists within the esophagus but goes unnoticed when measurements are performed with a long ballon (often 10 cm in length).

End-expiratory pressure has previously been shown not to correlate to chest wall elastance [29] which is in line with our findings with increased tidal variation of esophageal pressure, interpreted as increased chest wall elastance, but lower end-expiratory esophageal pressure in prone position. The lower PES_EE_ seen in prone position has been described before, using an esophageal balloon [30, 31] and probably reflects both a change in pleural pressure at that level as well as less weight load from the mediastinal organs. The difference in end-expiratory pressure between supine and prone position is in line with other studies using a ballon catheter both in patients and in a porcine model even if the absolute values differ [15, 17]. The observed difference in absolute pressures disappears when only selected sensors are included (PES_OPT_), which indicates that the higher end-expiratory pressures in our study is caused by local high esophageal pressures affecting the mean. Notably the difference between supine and prone position is lower compared to the difference between sitting and supine in our previous study [10]. The influence from the heart on esophageal measurements (cardiac oscillations) are often used as a marker of correct balloon positioning [32]. Cardiac oscillations are clearly visible and easy to quantify when HRM is used and were noted in all our patients. They were more pronounced in supine compared to prone position and in contrast to prone position cardiac oscillations decreased in supine when PEEP was increased. Studies in pigs and humans with ARDS has shown that center of ventilation is moved dorsally in supine and ventrally in prone in response to higher PEEP [33, 34]. Less cardiac oscillations at higher PEEP in supine position could possibly be explained with a PEEP-induced offloading of the heart when dorsal lung regions are inflated.

### Optimal esophageal pressure

The calculation of PES_OPT_ is an effort to circumvent the variability in esophageal pressure within a person by utilizing the possibilities with HRM, to select local esophageal pressures more likely to be representative of pleural pressure. Several studies have suggested that esophageal pressure overestimates pleural pressure [15, 35] and a correction factor has been proposed [36] for patients in supine position. The purpose of selecting pressure channels with the lowest end-expiratory esophageal pressures, is to mitigate the risk of overestimating pleural pressure with esophageal pressure measurements [37]. When using only the lowest absolute esophageal pressures in the lower part of esophagus (PES_OPT_) the mean pressure is 5–7 cmH_2_O lower than the average of all pressures in the part of esophagus used for balloon measurements, which corresponds to the correction factor suggested by other researchers [11, 36]. Sensors included in PES_OPT_ varied between each patient and no clear pattern was obvious which makes it difficult to predict where an optimal esophageal pressure can be measured, see figure S9 in supplementary material.

### Tidal variation of esophageal pressure

Also, the tidal variation of esophageal pressure is highly variable with 90% of patients having a negative ΔPES on at least one level of the esophagus in supine and/or prone. Negative changes in esophageal pressure unlikely represents changes in pleural pressure during controlled inflation in relaxed patients, but instead a local off-loading of an external weight by lung inflation. Local negative ΔPES would go undetected if measurements were performed using an esophageal balloon with no need of a physiological interpretation of a negative pressure swing during controlled inflation. The localized negative ΔPES contributes to the very high coefficient of variation in Table 3 (5.4 at PEEP 5 in supine position). Because of both positive and negative ΔPES the mean ΔPES within some patients is close to zero which results in a very high coefficient of variation without an unusually high within-patient SD.

### Clinical implications

Esophageal pressures have been evaluated extensively over the years with gradually increased understanding of its physiology. Still how to use the pressure in the clinical context is under discussion [3]. The findings in the present study adds further knowledge about the complexity of esophageal pressure and the difficulty to use a single esophageal pressure, estimated with a balloon catheter or a single tip manometry catheter, as a substitute for pleural pressure at the bedside. Selection of “optimal” esophageal pressure measurements with a HRM catheter is theoretically appealing to provide a more representative substrate for pleural pressure but its use is limited to research situations since introducing HRM in clinical practice in the ICU is not realistic for several reasons. From our data it is also not possible to make recommendations about how to obtain optimal esophageal pressures with a balloon catheter in a clinical situation. HRM measurements on the other hand indicates that the single pressure presented from a regular ballon catheter at the bedside, is the results of many highly variable esophageal pressures affecting a semi-filled often 10 cm long balloon. The degree of variation, likely due to multiple factors, is unknown to the clinician and the measured esophageal pressure from a balloon catheter should be interpreted with these factors in mind. The lack of strong evidence for clinical benefit of esophageal pressure measurements is likely explained by the multiple factors influencing the pressure which makes it difficult to use measurements from just one region of the esophagus as basis for ventilator settings.

## Conclusion

In both supine and prone position esophageal pressures are highly variable within a patient but to different degrees affected by change in ventilator settings and external factors from the mediastinal organs.

Statements and Declarations.

## Supplementary Information

Below is the link to the electronic supplementary material.

Supplementary Material 1

## Data Availability

No datasets were generated or analysed during the current study.
