# Supplementary material for: Esophageal pressures in supine and prone position: evaluation using high-resolution manometry
Source: J Clin Monit Comput. 2026 Jun 11;40(5):1177–88. doi: 10.1007/s10877-026-01456-6 (PMC13562092; doi:10.1007/s10877-026-01456-6)
Supplement: Supplementary file 1 [file 10877_2026_1456_MOESM1_ESM.pdf]

Age (years)	Sex	Height (m)	Weight (kg)	BMI (kg/m2)	IBW (kg)	ASA
**73**	F	1.6	60	23.4	52	2
**65**	F	1.69	91	31.9	61	2
**62**	M	1.73	80	26.7	69	2
**62**	M	1.75	100	32.7	70	2
**57**	F	1.71	64	21.9	62	1
**76**	M	1.87	88	25.2	81	2
**21**	F	1.62	60	22.9	54	1
**57**	M	1.75	80	26.1	70	2
**53**	F	1.63	92	34.6	55	2
**61**	M	1.79	85	26.5	74	2
**68**	M	1.9	84	23.3	84	2
**71**	M	1.76	80	25.8	71	3
**66**	M	1.78	78	24.6	73	3
**76**	M	1.84	100	29.5	81	3
**59**	F	1.72	80	27	63	2
**56**	F	1.61	100	38.6	53	2
**39**	F	1.7	102	35.3	61	2
**17**	F	1.66	57	20.7	58	1
**33**	F	1.76	75	24.2	67	1
**42**	F	1.8	94	29	70	1

Table S1. Patient characteristics.

Patient no	VT (ml)	VT (ml/kg)	PEEP 1 (cmH2O)	PPLAT 1 (cmH2O)	PEEP 2 (cmH2O)	PPLAT 2 (cmH2O)
**1**	320	6.15	5	11	12	16
**2**	370	6.07	5	10	12	16
**3**	420	6.09	5	12	12	18
**4**	420	6.00	5	16	12	21
**5**	370	5.97	5	12	12	20
**6**	490	6.00	5.4	11.5	12.2	18.5
**7**	330	5.81	4	11.4	11.9	17.5
**8**	420	6.00	5	13	12	20
**9**	330	6,00	5	13	12	19
**10**	450	6.08	5	12	12	20
**11**	500	5.93	5.4	12.4	12.4	19.1
**12**	430	6.06	5	12	12	19
**13**	440	6.00	5	13	12	19
**14**	490	6.02	5.3	15.7	12.3	22.5
**15**	380	5.95	5.3	12.5	12.3	19.1
**16**	320	6.02	5.8	16.5	12.9	22.5
**17**	370	6.03	5.4	15.2	12.4	22.7
**18**	350	6.00	5.4	10	12.4	16.5
**19**	400	5.93	5.4	11.3	12.3	18
**20**	420	5.94	5.2	13.7	12.2	20
**Mean**	398.9	6.0	5.1	12.7	12.2	19.2
**SD**	57.3	0.1	0.3	1.9	0.2	2

Table S2. Respiratory data Supine

	Coefficient of variation 22-44cm			
	PES_{EE}		ΔPES	
	Supine<br>Mean (SD)<br>Median (min-max)	Prone<br>Mean (SD)<br>Median (min-max)	Supine<br>Mean (SD)<br>Median (min-max)	Prone<br>Mean (SD)<br>Median (min-max)
PEEP 5	0.74 (0.3)<br>0.64 (0.4-1.4)	1.77 (1.0)<br>1.2 (0.3-6.6)	2.44 (2.5)<br>1.33 (0.5-10.3)	3.27 (3.9)<br>0.95 (0.6-10.9)
PEEP 12	0.69 (0.4)<br>0.6 (0.2-1.4)	1.18 (1.5)<br>1.13 (0.3-5.4)	1.6 (0.8)<br>1.00 (0.3-4.1)	4.19 (6.5)<br>1.29 (0.6-18)

Table S3. Coefficient of variation within patients, for total esophagus (22-44cm).

	End-expiratory $PES_{30\text{-}42cm}$ and PES_{opt}					
	Supine			Prone		
	$PES_{30\text{-}42cm}$<br>Mean (SD)	PES_{opt}<br>Mean (SD)	Difference<br>Mean-Mean<br>(95% CI)	$PES_{30\text{-}42cm}$<br>Mean (SD)	PES_{opt}<br>Mean (SD)	Difference<br>Mean-Mean<br>(95% CI)
PEEP 5	15.6 (8.7)	9.5 (7.6)	6.1<br>(4.0; 8.2)*	10.2 (11.5)	4.2 (8.8)	6.0<br>(2.7; 9.3)*
PEEP 12	16.5 (8.6)	9.9 (7.8)	6.6<br>(4.1; 9.0)*	11.9 (10.9)	6.7 (10.0)	5.3<br>(2.4; 8.1)*

Table S4. Comparing end-expiratory PES. *Confidence interval separated from 0

	Coefficient of variation PES_{OPT}			
	PES_{EE}		ΔPES	
	Supine<br>Mean (SD)<br>Median (min-max)	Prone<br>Mean (SD)<br>Median (min-max)	Supine<br>Mean (SD)<br>Median (min-max)	Prone<br>Mean (SD)<br>Median (min-max)
PEEP 5	0.96 (1.9)<br>0.43 (0.1; 8.8)	1.06 (0.9)<br>1.04 (0; 2.9)	0.55 (0.4)<br>0.47 (0.1; 1.6)	0.38 (0.4)<br>0.4 (0; 1.6)
PEEP 12	0.93 (1.4)<br>0.3 (0.1; 4.7)	0.82 (1.3)<br>0.33 (0; 5.5)	0.53 (0.36)<br>0.49 (0.1; 1.4)	0.43 (0.3)<br>0.4 (0; 1.2)

Table S5. Coefficient of variation within patients, for optimal PES.

	PES_{EE} PEEP 5 Supine	PES_{EE} PEEP 12 Supine	ΔPES PEEP 5 Supine	ΔPES PEEP 12 Supine	PES_{EE} PEEP 5 Prone	PES_{EE} PEEP 12 Prone	ΔPES PEEP 5 Prone	ΔPES PEEP 12 Prone
22-44cm	0.5	0.48	0.54	0.46	1.01	0.87	0.91	0.87
30-42cm	0.56	0.53	0.82	0.71	1.13	0.91	0.84	0.84
PES_{OPT}	0.8	0.79	0.57	0.6	2.09	1.5	0.6	0.62

Table S6. Coefficient of variation between patients.

	Supine		Prone	
	$PES_{30\text{-}42cm}$	PES_{OPT}	$PES_{30\text{-}42cm}$	PES_{OPT}
Max-Min	25.0	7.8	21.9	5.8
SD	8.9	4.1	8.0	3.2

Table S7. Average difference between maximal and minimal PES_{EE} and mean SD of PES_{EE} within patients. PES_{EE} in cmH_2O with PEEP 5.

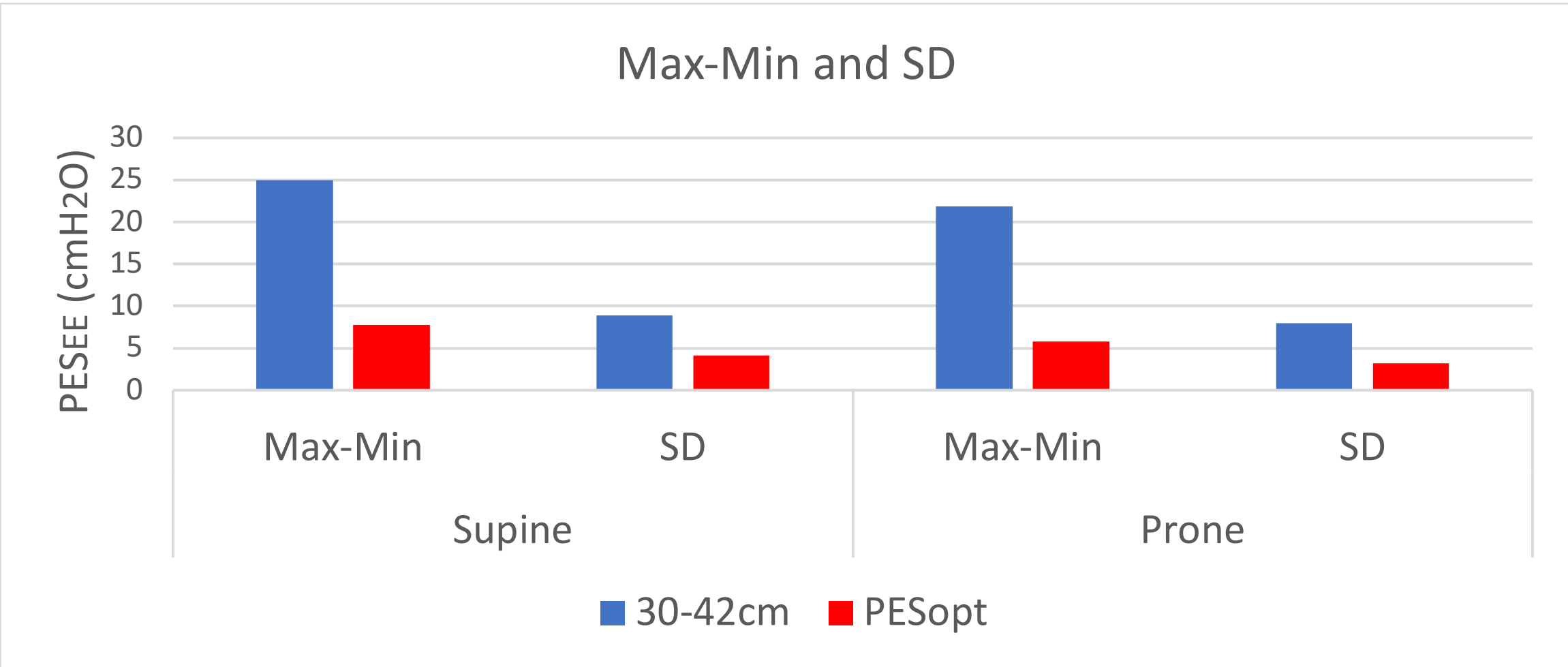

Figure S1. Average difference between maximal and minimal PES_{EE} and mean SD of PES_{EE} within patients. PES_{EE} in cmH_2O in supine and prone with PEEP 5.

	ΔPES/ΔPAW Mean (SD)
22-44cm	0.89 (0.44)
30-42cm	1.08 (0.44)
PES_{OPT}	1.12 (0.37)

Table S8. ΔPES/ΔPAW-ratio. During Baydur maneuver in pressure support ventilation.

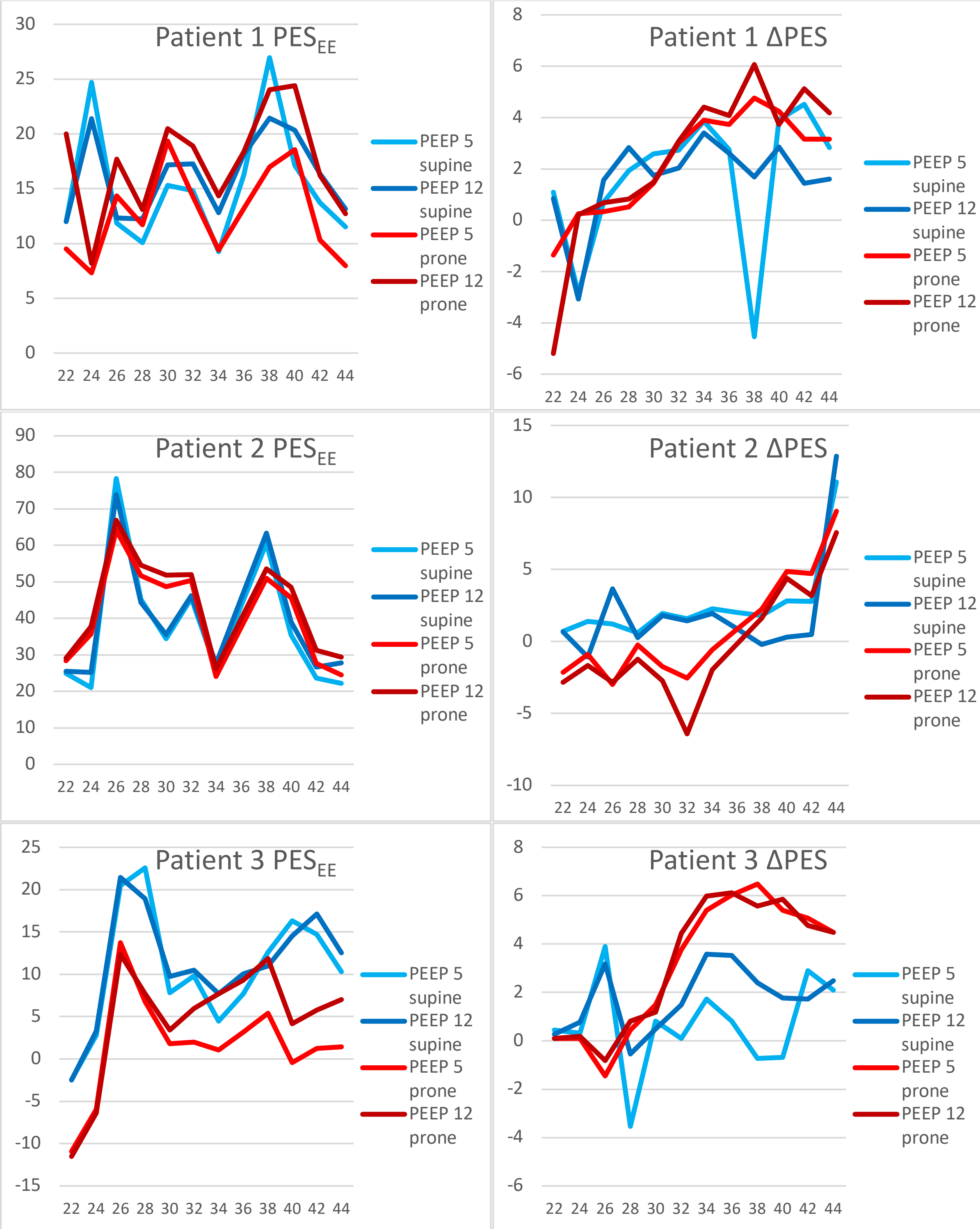
Patient 1 PES_{EE}
30
25
20
15
10
5
0
22 24 26 28 30 32 34 36 38 40 42 44
PEEP 5 supine
PEEP 12 supine
PEEP 5 prone
PEEP 12 prone
Patient 1 ΔPES
8
6
4
2
0
-2
-4
-6
22 24 26 28 30 32 34 36 38 40 42 44
PEEP 5 supine
PEEP 12 supine
PEEP 5 prone
PEEP 12 prone
Patient 2 PES_{EE}
90
80
70
60
50
40
30
20
10
0
22 24 26 28 30 32 34 36 38 40 42 44
PEEP 5 supine
PEEP 12 supine
PEEP 5 prone
PEEP 12 prone
Patient 2 ΔPES
15
10
5
0
-5
-10
22 24 26 28 30 32 34 36 38 40 42 44
PEEP 5 supine
PEEP 12 supine
PEEP 5 prone
PEEP 12 prone
Patient 3 PES_{EE}
25
20
15
10
5
0
-5
-10
-15
22 24 26 28 30 32 34 36 38 40 42 44
PEEP 5 supine
PEEP 12 supine
PEEP 5 prone
PEEP 12 prone
Patient 3 ΔPES
8
6
4
2
0
-2
-4
-6
22 24 26 28 30 32 34 36 38 40 42 44
PEEP 5 supine
PEEP 12 supine
PEEP 5 prone
PEEP 12 prone

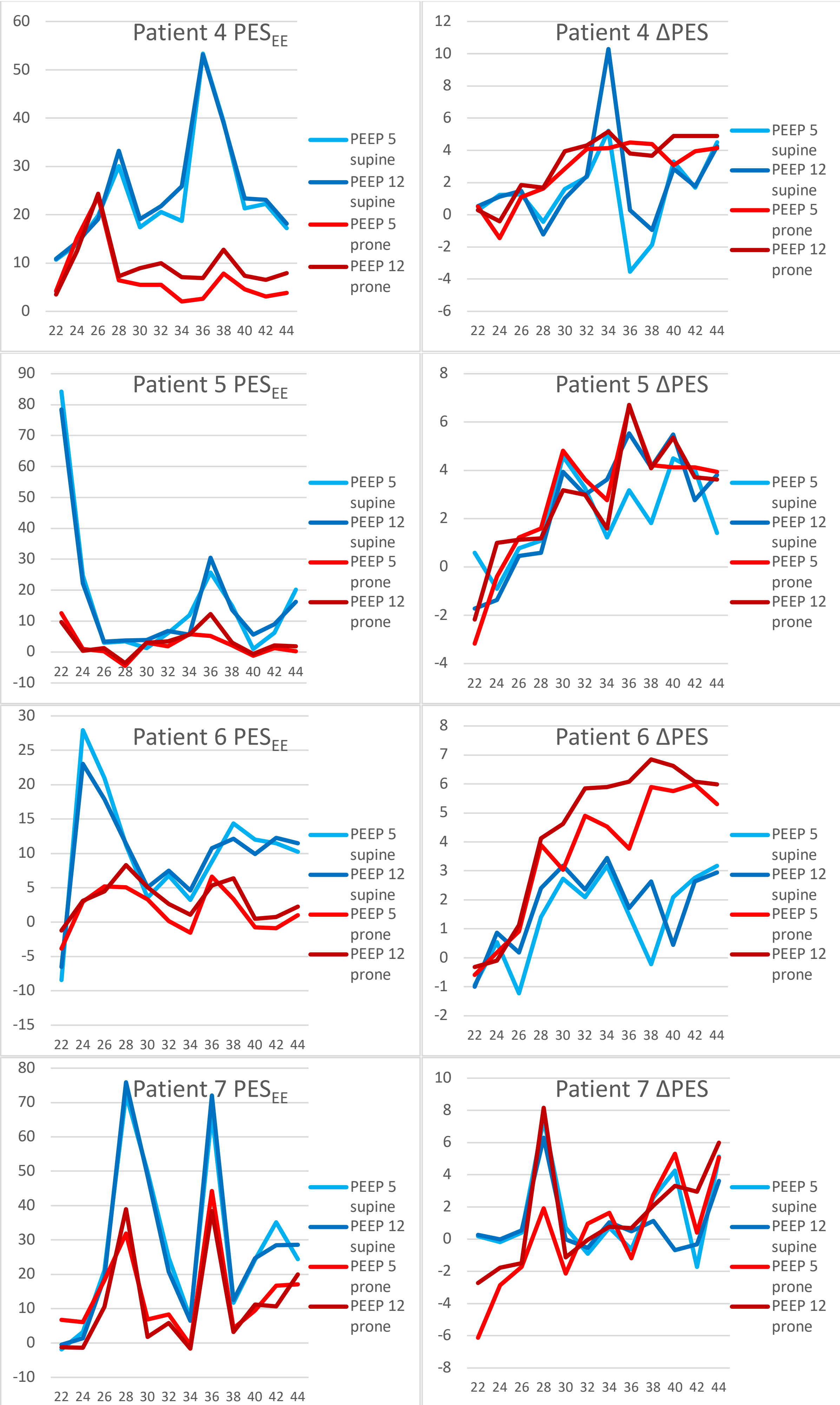
Patient 4 PES_{EE}
60
50
40
30
20
10
0
22 24 26 28 30 32 34 36 38 40 42 44
PEEP 5 supine
PEEP 12 supine
PEEP 5 prone
PEEP 12 prone
Patient 4 ΔPES
12
10
8
6
4
2
0
-2
-4
-6
22 24 26 28 30 32 34 36 38 40 42 44
PEEP 5 supine
PEEP 12 supine
PEEP 5 prone
PEEP 12 prone
Patient 5 PES_{EE}
90
80
70
60
50
40
30
20
10
0
-10
22 24 26 28 30 32 34 36 38 40 42 44
PEEP 5 supine
PEEP 12 supine
PEEP 5 prone
PEEP 12 prone
Patient 5 ΔPES
8
6
4
2
0
-2
-4
22 24 26 28 30 32 34 36 38 40 42 44
PEEP 5 supine
PEEP 12 supine
PEEP 5 prone
PEEP 12 prone
Patient 6 PES_{EE}
30
25
20
15
10
5
0
-5
-10
-15
22 24 26 28 30 32 34 36 38 40 42 44
PEEP 5 supine
PEEP 12 supine
PEEP 5 prone
PEEP 12 prone
Patient 6 ΔPES
8
7
6
5
4
3
2
1
0
-1
-2
22 24 26 28 30 32 34 36 38 40 42 44
PEEP 5 supine
PEEP 12 supine
PEEP 5 prone
PEEP 12 prone
Patient 7 PES_{EE}
80
70
60
50
40
30
20
10
0
-10
22 24 26 28 30 32 34 36 38 40 42 44
PEEP 5 supine
PEEP 12 supine
PEEP 5 prone
PEEP 12 prone
Patient 7 ΔPES
10
8
6
4
2
0
-2
-4
-6
-8
22 24 26 28 30 32 34 36 38 40 42 44
PEEP 5 supine
PEEP 12 supine
PEEP 5 prone
PEEP 12 prone

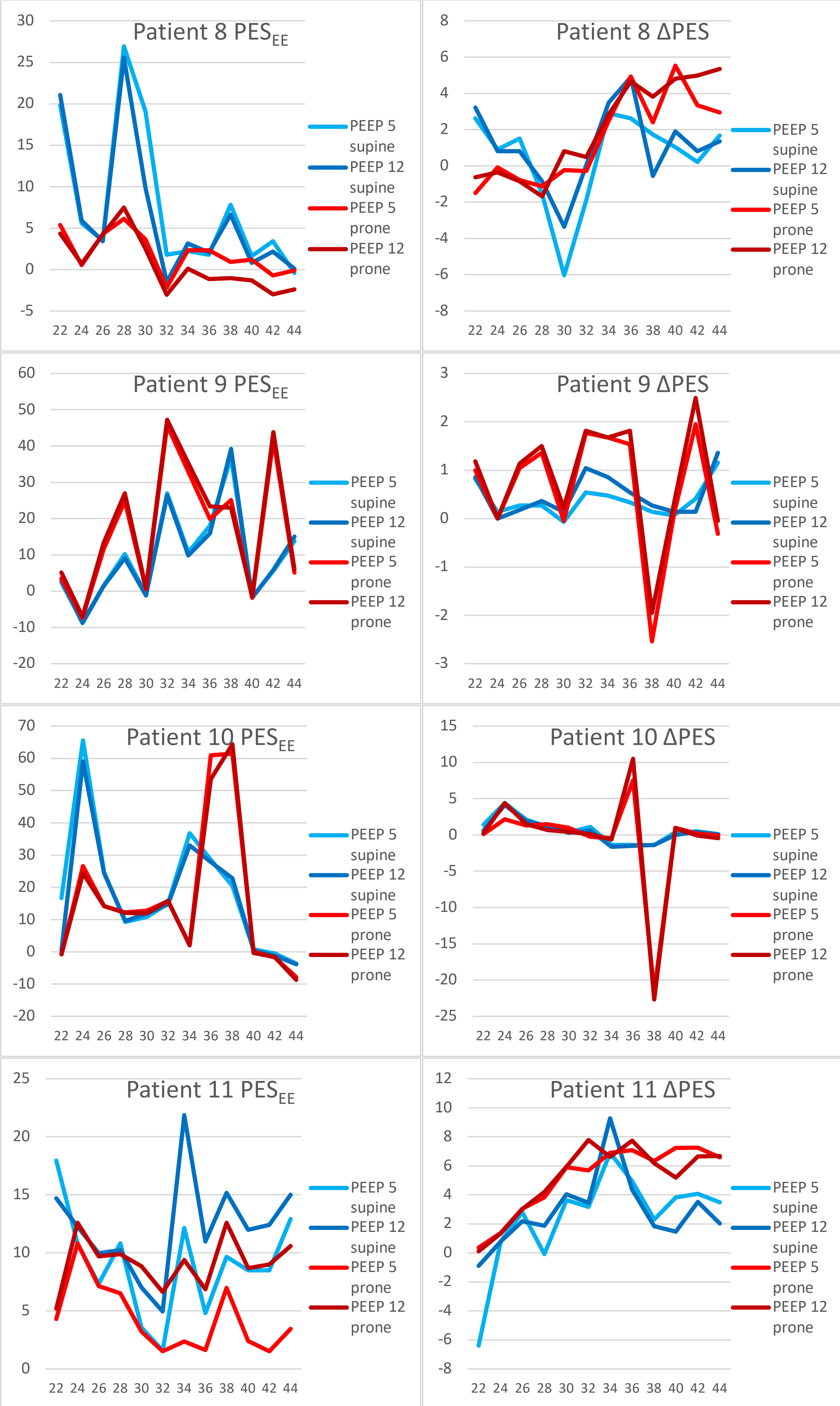
Patient 8 PES$_{EE}$
Patient 8 ΔPES
Patient 9 PES$_{EE}$
Patient 9 ΔPES
Patient 10 PES$_{EE}$
Patient 10 ΔPES
Patient 11 PES$_{EE}$
Patient 11 ΔPES
PEEP 5 supine
PEEP 12 supine
PEEP 5 prone
PEEP 12 prone
22 24 26 28 30 32 34 36 38 40 42 44

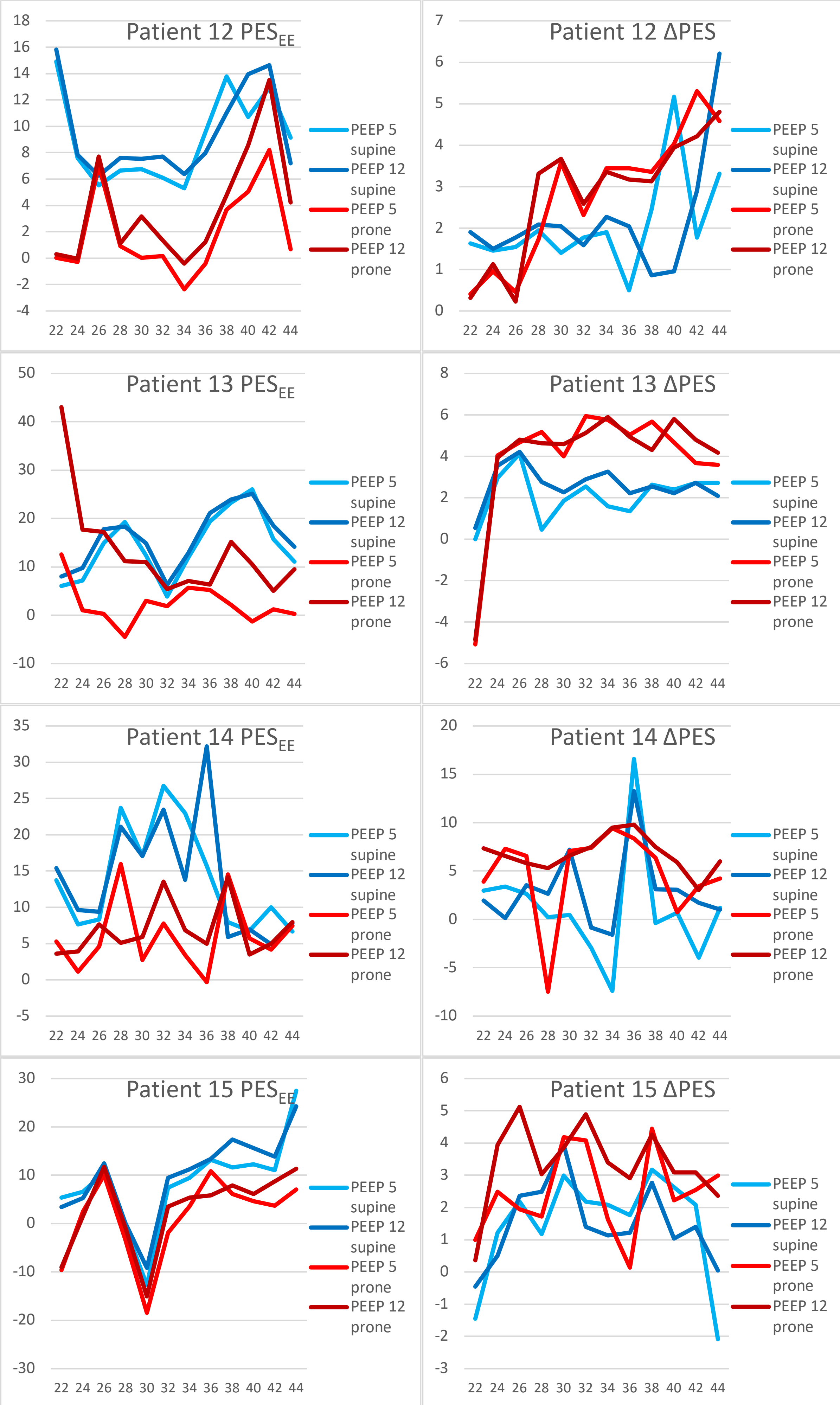

Patient 12 PES_{EE}
18
16
14
12
10
8
6
4
2
0
-2
-4
22 24 26 28 30 32 34 36 38 40 42 44
PEEP 5 supine
PEEP 12 supine
PEEP 5 prone
PEEP 12 prone
Patient 12 ΔPES
7
6
5
4
3
2
1
0
22 24 26 28 30 32 34 36 38 40 42 44
PEEP 5 supine
PEEP 12 supine
PEEP 5 prone
PEEP 12 prone
Patient 13 PES_{EE}
50
40
30
20
10
0
-10
22 24 26 28 30 32 34 36 38 40 42 44
PEEP 5 supine
PEEP 12 supine
PEEP 5 prone
PEEP 12 prone
Patient 13 ΔPES
8
6
4
2
0
-2
-4
-6
22 24 26 28 30 32 34 36 38 40 42 44
PEEP 5 supine
PEEP 12 supine
PEEP 5 prone
PEEP 12 prone
Patient 14 PES_{EE}
35
30
25
20
15
10
5
0
-5
22 24 26 28 30 32 34 36 38 40 42 44
PEEP 5 supine
PEEP 12 supine
PEEP 5 prone
PEEP 12 prone
Patient 14 ΔPES
20
15
10
5
0
-5
-10
22 24 26 28 30 32 34 36 38 40 42 44
PEEP 5 supine
PEEP 12 supine
PEEP 5 prone
PEEP 12 prone
Patient 15 PES_{EE}
30
20
10
0
-10
-20
-30
22 24 26 28 30 32 34 36 38 40 42 44
PEEP 5 supine
PEEP 12 supine
PEEP 5 prone
PEEP 12 prone
Patient 15 ΔPES
6
5
4
3
2
1
0
-1
-2
-3
22 24 26 28 30 32 34 36 38 40 42 44
PEEP 5 supine
PEEP 12 supine
PEEP 5 prone
PEEP 12 prone

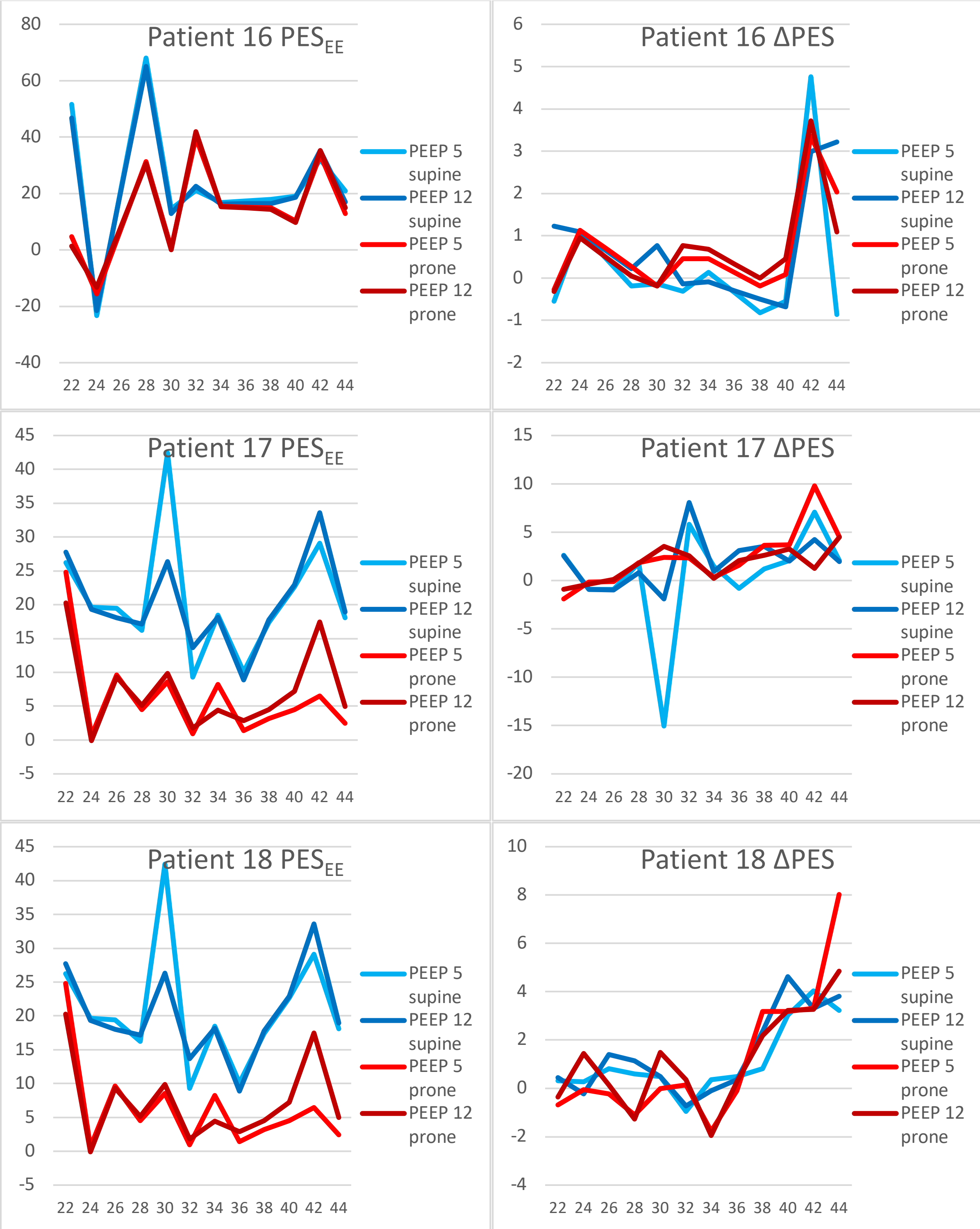
Patient 16 PES$_{EE}$
80
60
40
20
0
-20
-40
22 24 26 28 30 32 34 36 38 40 42 44
PEEP 5 supine
PEEP 12 supine
PEEP 5 prone
PEEP 12 prone
Patient 16 ΔPES
6
5
4
3
2
1
0
-1
-2
22 24 26 28 30 32 34 36 38 40 42 44
PEEP 5 supine
PEEP 12 supine
PEEP 5 prone
PEEP 12 prone
Patient 17 PES$_{EE}$
45
40
35
30
25
20
15
10
5
0
-5
22 24 26 28 30 32 34 36 38 40 42 44
PEEP 5 supine
PEEP 12 supine
PEEP 5 prone
PEEP 12 prone
Patient 17 ΔPES
15
10
5
0
-5
-10
-15
-20
22 24 26 28 30 32 34 36 38 40 42 44
PEEP 5 supine
PEEP 12 supine
PEEP 5 prone
PEEP 12 prone
Patient 18 PES$_{EE}$
45
40
35
30
25
20
15
10
5
0
-5
22 24 26 28 30 32 34 36 38 40 42 44
PEEP 5 supine
PEEP 12 supine
PEEP 5 prone
PEEP 12 prone
Patient 18 ΔPES
10
8
6
4
2
0
-2
-4
22 24 26 28 30 32 34 36 38 40 42 44
PEEP 5 supine
PEEP 12 supine
PEEP 5 prone
PEEP 12 prone

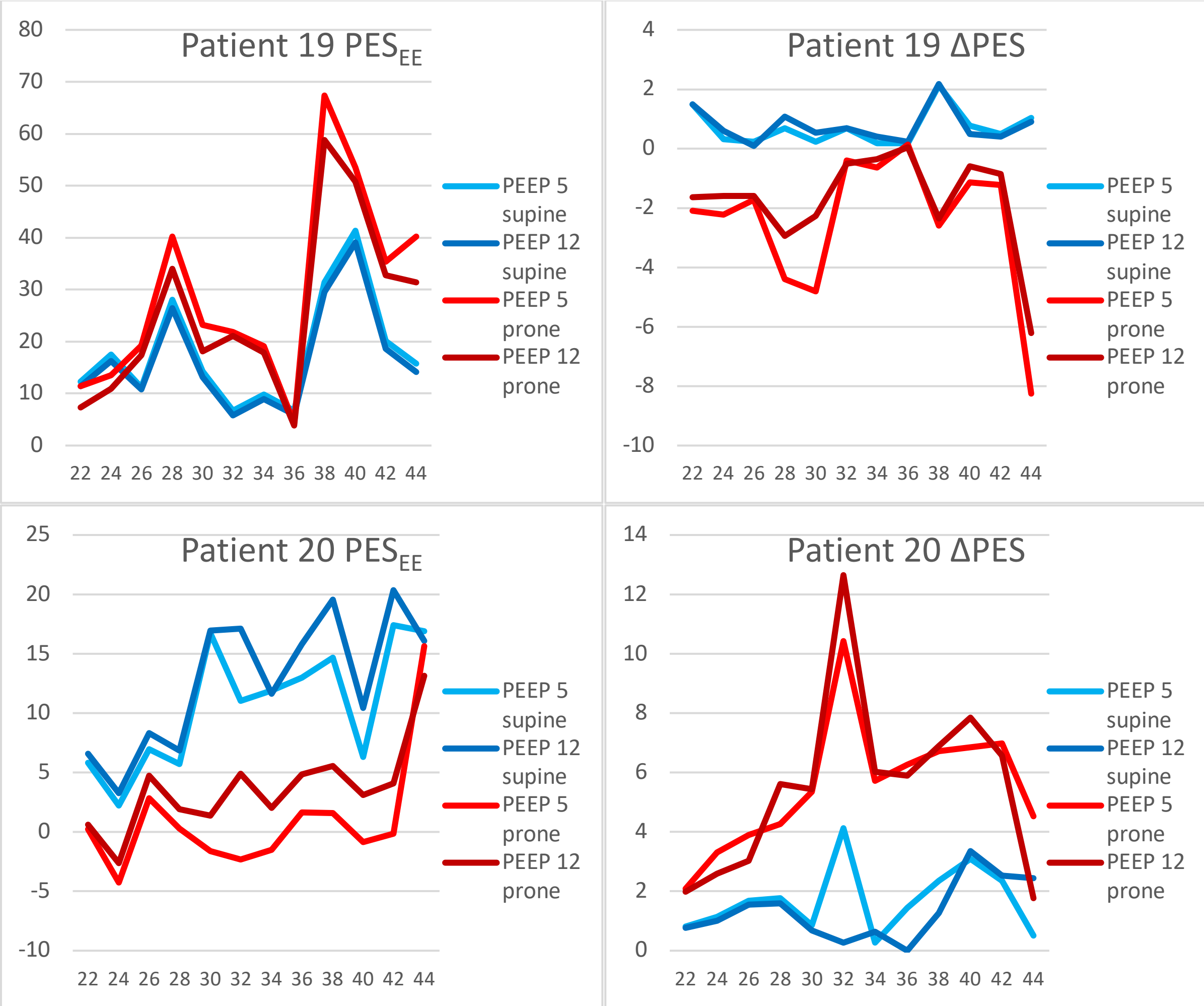

Figure S2-41. Left column all individual patients PES$_{end\text{-}expiratory}$. Right column all individual patients ΔPES. Pressure in cmH_2O on the Y-axis. X-axis show distance from the nostril in cm.

	1	2	3	4	5	6	7	8	9	10	11	12	13	14	15	16	17	18	19	20
**Selected sensors (cm)**	32<br>34<br>42	30<br>34<br>42	30<br>34<br>36	30<br>32<br>34	30<br>32<br>40	30<br>32<br>34	34<br>38<br>40	34<br>36<br>40	34<br>40<br>42	30<br>40<br>42	30<br>32<br>36	30<br>32<br>34	30<br>32<br>34	30<br>36<br>40	30<br>32<br>34	34<br>42	32<br>34<br>38	30<br>38<br>40	32<br>34<br>36	32<br>34<br>40

Table S9. Sensors included in PES$_{OPT}$ in each patient

Patient	Sex (M/F)	Weight (kg)	Length (m)	BMI (kg/m^2)	Diagnosis	FiO_2	PEEP	PS
**1**	M	68	1,8	21	Encephalitis	0,25	10	8
**2**	M	63	1,84	19	Pneumonia	0,3	8	8
**3**	M	95	1,85	28	Cardiac arrest	0,3	12	11
**4**	M	68	1,74	22	Burns	0,45	8	8
**5**	M	76	1,72	26	Covid-19	0,4	8	8
**6**	M	102	1,72	34	Postoperative bleeding	0,3	12	10
**7**	M	66	1,7	23	Traumatic brain injury	0,3	10	8
**8**	M	121	1,88	34	Cardiac arrest	0,45	10	8
**9**	F	100	1,64	37	Cardiac arrest	0,25	12	10
**10**	M	107	1,85	31	Postoperative bleeding	0,35	10	8

Table S10. Patient characteristics in separate cohort.
BMI=body mass index, FiO_2= fraction of inspired oxygen, PEEP= positive end-expiratory pressure, PS= pressure support
